# Humulus Lupulus. Hop.

**Published:** 1859-02

**Authors:** 


*Humulus Lupulus. Hop.*—Dr. Wilson, of Port Huron, states "That Lupulin is described in the books as a narcotic sedative. I have used it a great deal, but never saw anything approaching to narcotism produced by it. I have found it to be an efficient antiphrodisiac in spermatorrhœa. Dr. Smith of Troy, in this State, says he used it with considerable benefit in a case of hysteria, but a tolerance was soon established, and after a few days it had no more effect. I have observed this, but generally found that if, after a suspension of a few days, it be again used, it is as efficient as ever. From comparing my experience with that of Dr. Smith, I would be inclined to regard it as having specific action upon the lower part of the spinal cord, depressing its reflex power.—*Peninsular and Independent Medical Journal.*

